# Fine-tuning a blunt tool: Regulation of viral host shutoff RNases

**DOI:** 10.1371/journal.ppat.1008385

**Published:** 2020-04-08

**Authors:** Raecliffe Daly, Denys A. Khaperskyy, Marta Maria Gaglia

**Affiliations:** 1 Graduate Program in Cell, Molecular, and Developmental Biology, Graduate School of Biomedical Sciences, Tufts University, Boston, Massachusetts, United States of America; 2 Department of Molecular Biology and Microbiology, Tufts University School of Medicine, Boston, Massachusetts, United States of America; 3 Department of Microbiology & Immunology, Dalhousie University, Halifax, Nova Scotia, Canada; University of Wisconsin Madison, UNITED STATES

The evolutionary arms race between host and pathogen has resulted in the ability of many human viruses to alter the host gene expression profile during infection, in order to redirect cellular resources towards viral gene expression and inhibit cell-intrinsic host immune responses. In particular, multiple viruses globally reduce host gene expression in a process termed “host shutoff.” Multiple mechanisms of host shutoff exist, including translational and transcriptional shutoff, but several viruses carry out host shutoff by encoding ribonucleases (RNases) that degrade host messenger RNAs (mRNAs). Viral host shutoff RNases include the influenza A virus polymerase acidic-X (PA-X) [1], the herpes simplex viruses (HSV-1 and -2) virion host shutoff protein (vhs) [2], and the Kaposi’s sarcoma-associated herpesvirus (KSHV) shutoff and exonuclease (SOX) protein [3] and its homologs, muSOX from murine gammaherpesvirus 68 (MHV68) [4] and BGLF5 from Epstein–Barr virus (EBV) [5]. These RNases contribute to efficient formation of virions and/or reduction of innate immune signaling [6–8]. For example, in the absence of EBV BGLF5, the virus produces fewer mature capsids, many of which remain trapped in the nucleus [7]. Vhs-deficient HSV replicates well in many common tissue culture models [9] but shows replication defects in relevant cell types, such as cerebellar granule neurons [8]. Moreover, in mice, viruses lacking detectable host shutoff activity replicate to lower viral titers in neuronal tissue [9], indicating a restriction of viral replication probably related to host immune responses. Influenza A virus PA-X also limits host antiviral and proinflammatory responses in several animal models [1,10,11] but has minimal effect on viral replication both in vivo and in cell culture [1,11,12]. Although host shutoff RNases are important for successful viral infection, their activity presents an interesting problem for the viruses that encode them. Unregulated RNase activity could degrade viral RNAs or host mRNAs encoding proteins that the virus needs. Moreover, drastic depletion of the mRNA pool by the virus could trigger antiviral host stress responses and cell death. It is thus unsurprising that evidence is now emerging that viruses posttranslationally regulate the activity of their host shutoff RNases through a variety of mechanisms, reviewed herein (Fig 1), to fine-tune host gene regulation without inhibiting viral replication.

**Fig 1:**
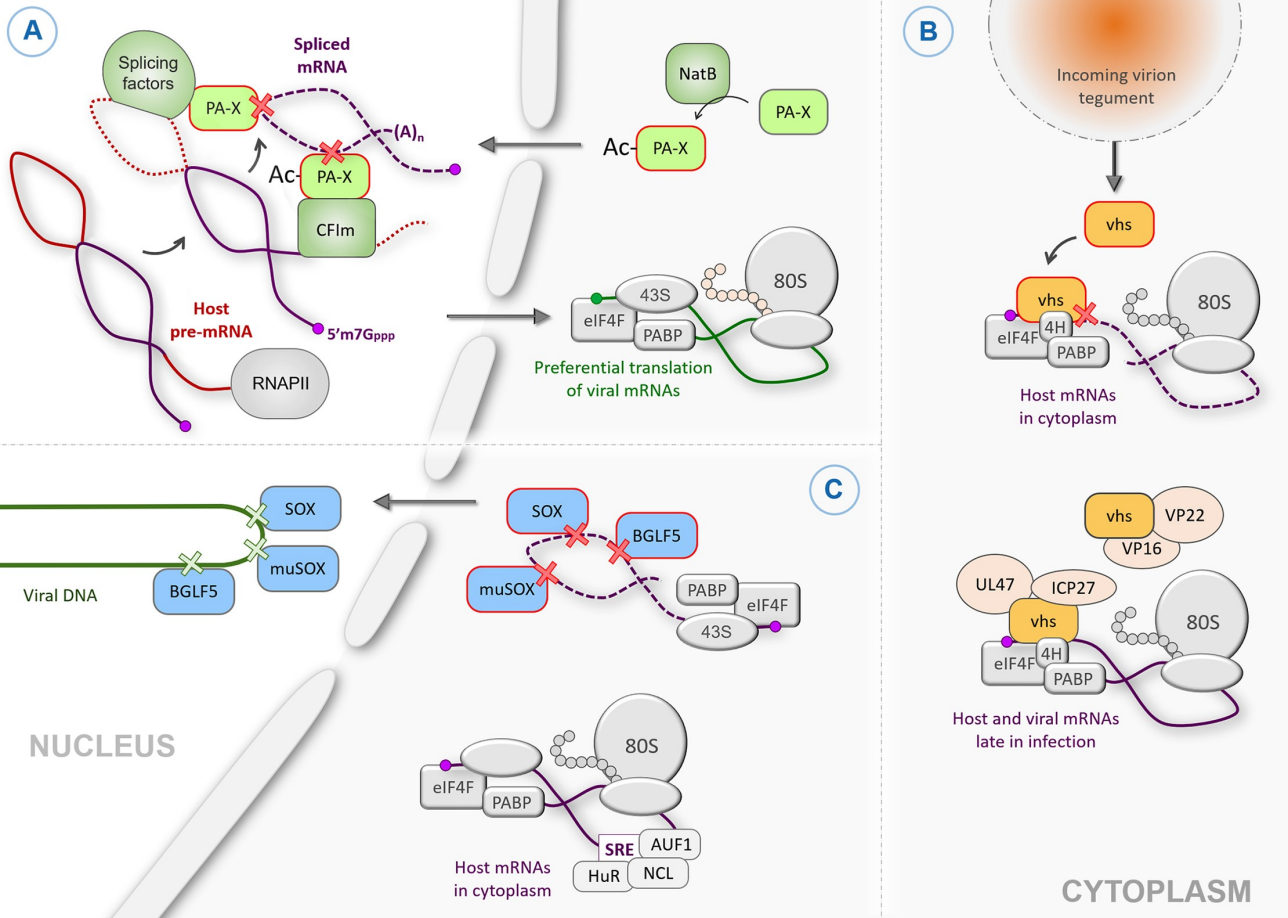
Different mechanisms of viral host shutoff nuclease regulation. (A) The influenza A virus endonuclease PA-X functions in the cell nucleus. In order to be fully active, nascent PA-X proteins have to be N-terminally acetylated (Ac-PA-X) by the host enzyme NatB. In the nucleus, PA-X associates with pre-mRNA processing factors, including splicing factors and the CFIm complex, which recruit PA-X to spliced transcripts. Unspliced viral mRNAs and host intronless mRNAs escape PA-X-mediated degradation and are translated in the cytoplasm. (B) Regulation of HSV-1 nuclease vhs through interactions with other viral proteins. As part of the virion, vhs is released into the cytoplasm upon infection, where it targets translation-competent host mRNAs through association with the components of the cap-binding complex eIF4F and the translation initiation factor eIF4H (4H). Late in infection, nuclease activity of the newly synthesized vhs is inhibited through interaction with viral proteins VP16, VP22, UL47, and ICP27. (C) The KSHV endonuclease SOX and its homologs muSOX and BGLF5 from the closely related herpesviruses MHV68 and EBV, respectively, are regulated through multiple mechanisms. In the cytoplasm of infected cells, SOX-like proteins preferentially cleave mRNAs, whereas in the nucleus, they function as DNases and help resolve concatemers of replicating viral DNA. Select host mRNAs escape SOX-mediated degradation by possessing protective SREs in their 3′ untranslated regions, which recruits cellular binding proteins, including HuR, AUF1, and NCL. In all panels, red outlines denote the host shutoff nucleases in their active forms. Ac-PA-X, acetylated PA-X; CFIm, cleavage factor Im; EBV, Epstein–Barr virus; eIF4H (4H), eukaryotic initiation factor 4H; HSV, herpes simplex virus; KSHV, Kaposi’s sarcoma-associated herpesvirus; mRNA, messenger RNA; NatB, N-acetyl transferase B; NCL, nucleolin; SRE, SOX resistance element; vhs, virion host shutoff protein.

## Selection of targeted and protected RNAs and protein/protein interactions

All host shutoff RNases cause global decreases in host mRNA abundance, revealed by transcriptome-wide studies [13–15], and display a preference for mRNAs while sparing housekeeping noncoding RNAs (ncRNAs) [12,16,17]. However, host shutoff RNases are less promiscuous than these results suggest and can select both for and against specific targets [12,13,15–20].

For influenza PA-X, the selectivity for mRNAs (and other transcripts of RNA polymerase II) is linked to RNA splicing, which is tightly connected to RNA polymerase II transcription [13]. Spliced mRNAs are more susceptible to PA-X than mRNAs that are naturally intronless or have been engineered to have no introns [13]. This selectivity may be due to direct physical interactions between PA-X and host proteins associated with splicing and other RNA processing [13]. The splicing-based targeting strategy may directly allow viral transcripts to be spared from PA-X-mediated degradation (Fig 1A). Influenza mRNAs are transcribed by the viral RNA-dependent RNA polymerase, not host machinery, and only 2 out of 8 genomic segments undergo splicing. Interestingly, we have demonstrated that these spliced influenza mRNAs are still protected from PA-X degradation [13], perhaps because their splicing does not require the same factors as host pre-mRNAs or simply because splicing of viral transcripts is inefficient.

In contrast, all the herpesviral nucleases selectively target mRNAs because they are actively translated [16,17]. HSV vhs selects translating mRNAs by directly interacting with the cellular translation initiation factors [20,21] (Fig 1B). For KSHV SOX and its homologs, no specific cofactor has yet been identified, but the connection to translation is supported by experiments showing SOX cosediments with 40S ribosomal translation initiation complexes, indicating that mRNAs are targeted at an early step of translation [16]. Importantly, herpesviral mRNAs are not immune to the degradation, as the same translational machinery is utilized for both viral and host mRNAs [21,22]. How herpesviruses compensate for this apparent problem to maintain efficient viral gene expression remains unknown.

In an interesting case of convergent evolution, all the host shutoff RNases described so far are endoribonucleases and cut mRNAs in fragments rather than initiating degradation from an mRNA end [12,16,17,23–25]. This strategy may be preferable because it rapidly disables the mRNA and renders it untranslatable. Interestingly, cleavages by KSHV SOX appear to occur at specific sites in the mRNAs that are marked by a degenerate sequence motif and structural element [16,26,27]. It is still unclear whether there is any reason behind the sequence specificity and whether other host shutoff RNases have any sequence specificity. HSV vhs is thought to cleave most RNAs close to the 5′ cap, with the exception of stress response mRNAs with adenylate/uridylate (AU)-rich elements in their 3′ untranslated region (UTR), which vhs cleaves near the AU-rich element and whose 5′ portion remains stable [28,29]. Additional complexity to the SOX targeting mechanism was revealed by the identification of SOX-resistant, or “escapee” mRNAs, which contain a structured RNA element, the “SOX resistance element” (SRE), in the 3′ UTR [15,30,31]. The SRE recruits several host RNA binding proteins that inhibit SOX-mediated cleavage through an unknown mechanism (Fig 1C) [30–32]. Interestingly, the SRE also appears to protect mRNAs from degradation by PA-X and vhs, as well as the SOX homologs [31]. Overall, these studies show that host shutoff RNases have multiple levels of RNA selectivity that counteracts the apparent promiscuity of these enzymes.

## Modulation of host shutoff activity by other viral proteins

Multiple studies show that the activity of the HSV RNase vhs is modulated by other viral proteins. As the “virion host shutoff” name suggests, vhs is a component of the virion and inhibits gene expression immediately after release from the entering virions [2]. However, vhs is itself expressed with late kinetics for incorporation into nascent virions and is inactive at this stage of the replication cycle, suggesting it is actively inhibited [33,34]. Initially postulated by Fenwick and colleagues [35], the existence of HSV proteins that modulate vhs expression was demonstrated by the Jones, Baines, and Roizman groups. Indeed, 4 HSV proteins bind and inactivate vhs: virion protein (VP)16, VP22, UL47 (also known as VP13/14), and infected cell protein 27 (ICP27) [33,34] (Fig 1B). VP22 may also play a role in overcoming the vhs-induced retention of vhs mRNA in the nucleus, thus relieving the inhibition of vhs translation in cells [36].

In contrast to vhs, gamma-herpesviral RNase activity is not restricted to the early part of the replication cycle. Nonetheless, the EBV protein kinase BGLF4 has been proposed to inhibit activity of the EBV RNase BGLF5 [37]. The mechanism remains unknown. BGLF4 was proposed to phosphorylate BGLF5 by an in vitro screen [38], but this finding was not confirmed by mass spectrometry analysis of phosphorylated EBV proteins in Burkitt’s lymphoma cells [39]. Also, it is not clear whether the BGLF5 homologs SOX and muSOX are also regulated by the homologous KSHV and MHV68 kinases.

Why do these viruses encode both RNases and inhibitors of the RNases? As mentioned previously, herpesviral host shutoff RNases can degrade viral as well as cellular mRNAs. In fact, HSV vhs is thought to degrade early viral mRNAs and contribute to the switch between early and late herpesviral gene expression [40]. Moreover, the absence of EBV BGLF5 and MHV68 muSOX results in aberrant virion composition, likely due to viral protein overproduction [12,37,41]. Thus, the virus may have evolved to use these RNases and their inhibitors to fine-tune not only cellular but also viral gene expression [42].

## Correct subcellular localization is key for host shutoff RNase function

vhs is primarily located in the cytoplasm [22], whereas PA-X and the gammaherpesviral RNases are primarily localized to the nucleus, with a portion found in the cytoplasm [4–6,12,43]. Nonetheless, for SOX and muSOX (and presumably BGLF5), it appears that the small cytoplasmic portion is required for host shutoff activity, consistent with their link to translation (Fig 1C). Indeed, trapping muSOX in the nucleus with a nuclear retention signal blocks host shutoff [4], whereas mutating the nuclear localization signal on SOX to make it more cytoplasmic does not [6]. The nuclear fraction of these proteins is likely used for a separate function of these enzymes in viral DNA processing [6,44] (Fig 1C). Because this genome processing function is conserved in all herpesviruses, including alpha-herpesviruses like HSVs [3,6,7,44], it appears that in alpha-herpesviruses, host shutoff and genome processing are separated by both localization and active factor, whereas in gamma-herpesviruses localization is the key determinant. In contrast, although influenza PA-X has a similar localization to SOX, the nuclear pool of PA-X appears to be the one important for function, as C-terminal truncations and mutations that abolish nuclear localization also reduce or abolish function [12,43].

Importantly, the functionally relevant localization of the RNases matches well with that of their cellular cofactors. For example, vhs-interacting proteins are cytoplasmic proteins involved in translation initiation, whereas PA-X-interacting proteins are nuclear proteins involved in RNA processing. Thus, correct localization presumably allows the RNases to interact with the correct cellular proteins and degrade the intended RNA targets.

## Co- and posttranslational modification of host shutoff RNases

Eukaryotic proteins commonly undergo co- and posttranslational modifications, such as phosphorylation, ubiquitination, and acetylation, which can regulate protein localization, stability, and function. So far, few modifications have been identified on host shutoff RNases. Although 3 differentially phosphorylated forms of HSV-1 vhs have been identified [45], it is unclear whether these phosphorylations alter vhs activity. As mentioned previously, BGLF5 appeared to be a substrate for BGLF4 phosphorylation in vitro [38], but whether this happens in vivo remains unclear. A less well-studied modification, N-terminal (Nt-) acetylation, has been described on PA-X (Fig 1A). Nt-acetylation occurs cotranslationally on 80% of all proteins and may play roles in subcellular localization, protein stability, and protein–protein interactions (reviewed in [46]). A recent report revealed that PA-X Nt-acetylation is required for its activity [47]. Interestingly, influenza PA-X appears to require acetylation specifically by 1 of the 6 human N-terminal acetylase (Nat) complexes, NatB, as PA-X mutants that are not recognized by NatB but are still acetylated (presumably by other Nats) have reduced host shutoff activity [47]. It remains possible that future studies will reveal additional relevant modifications on host shutoff RNases.

## Conclusion

Host shutoff is a key feature of many viral replication cycles that profoundly alters the host gene expression profile. It plays important roles in viral pathogenesis by suppressing host immune responses and redirecting cellular resources to viral gene expression. This warrants the detailed understanding of host shutoff mechanisms and regulation in different viruses, especially because the expression of viral mRNAs and key host mRNAs and/or ncRNAs must be preserved for replication to occur. Particularly, determining the host and viral interacting proteins of the RNases and characterizing the functional consequences of these interactions will shed new light on their target selection mechanism and wider roles in viral replication. Together with a better characterization of posttranslational modifications, it will give us a better understanding of the regulation of these potent viral factors and provide new targets for potential therapeutic interventions.
